# Finite element analysis comparing a PEEK posterior fixation device versus pedicle screws for lumbar fusion

**DOI:** 10.1186/s13018-023-04349-5

**Published:** 2023-11-10

**Authors:** Robert K. Eastlack, Pierce D. Nunley, Kornelis A. Poelstra, Alexander R. Vaccaro, Marcus Stone, Larry E. Miller, Pierre Legay, Julien Clin, Aakash Agarwal

**Affiliations:** 1 Department of Orthopaedic Surgery, Scripps Clinic, San Diego, CA USA; 2 Spine Institute of Louisiana, Shreveport, LA USA; 3 Department of Orthopaedic Surgery, Rothman Institute, Thomas Jefferson University, Philadelphia, PA USA; 4 Miller Scientific, 3101 Browns Mill Road, Ste 6, #311, Johnson City, TN 37604 USA; 5 Numalogics Inc., Montreal, QC Canada; 6 Departments of Bioengineering and Orthopaedic Surgery, University of Toledo, Toledo, OH USA

**Keywords:** Karma, Posterior fixation, Lumbar spine, Pedicle screw, Cortical fixation

## Abstract

**Background:**

Pedicle screw loosening and breakage are common causes of revision surgery after lumbar fusion. Thus, there remains a continued need for supplemental fixation options that offer immediate stability without the associated failure modes. This finite element analysis compared the biomechanical properties of a novel cortico-pedicular posterior fixation (CPPF) device with those of a conventional pedicle screw system (PSS).

**Methods:**

The CPPF device is a polyetheretherketone strap providing circumferential cortical fixation for lumbar fusion procedures via an arcuate tunnel. Using a validated finite element model, we compared the stability and load transfer characteristics of CPPF to intact conditions under a 415 N follower load and PSS conditions under a 222 N preload. Depending on the instrumented levels, two different interbody devices were used: a lateral lumbar interbody device at L4–5 or an anterior lumbar interbody device at L5-S1. Primary outcomes included range of motion of the functional spinal units and anterior load transfer, defined as the total load through the disk and interbody device after functional motion and follower load application.

**Results:**

Across all combinations of interbody devices and lumbar levels evaluated, CPPF consistently demonstrated significant reductions in flexion (ranging from 90 to 98%), extension (ranging from 88 to 94%), lateral bending (ranging from 75 to 80%), and torsion (ranging from 77 to 86%) compared to the intact spine. Stability provided by the CPPF device was comparable to PSS in all simulations (range of motion within 0.5 degrees for flexion–extension, 0.6 degrees for lateral bending, and 0.5 degrees for torsion). The total anterior load transfer was higher with CPPF versus PSS, with differences across all tested conditions ranging from 128 to 258 N during flexion, 89–323 N during extension, 135–377 N during lateral bending, 95–258 N during torsion, and 82–250 N during standing.

**Conclusion:**

Under the modeled conditions, cortico-pedicular fixation for supplementing anterior or lateral interbody devices between L4 and S1 resulted in comparable stability based on range of motion measures and less anterior column stress shielding based on total anterior load transfer measures compared to PSS. Clinical studies are needed to confirm these finite element analysis findings.

## Introduction

Anterior and lateral lumbar interbody fusion supplemented with posterior pedicle screws is a common construct for the operative management of many degenerative lumbar diseases. While standalone interbody cages can promote fusion, posterior stabilization provides additional biomechanical stability. Supplementing interbody fusion with posterior fixation has been shown to reduce range of motion, improve sagittal alignment, and provide higher fusion rates compared to interbody cages alone, typically with low complication rates [1, 2]. However, pedicle screw construct loosening and breakage may occur and are common causes for revision procedures [3]. These failures are often secondary to pseudarthrosis resulting from non-union, as continual stresses on a non-fused segment may lead to construct complications over time. The incidence of pedicle screw loosening in the literature varies considerably, generally ranging from 6 to 15% [4, 5]. The clinical course of patients with screw loosening is generally favorable. However, revision rates ranging from 1 to 5% are typical, with some studies reporting up to 16%, mainly due to screw-related chronic pain [5, 6]. These associated risks are higher in patients with low bone density [7]. Numerous attempts have been made to reduce these failure modes, such as the use of high-performance biomaterials, optimized design parameters, and augmentation of the screw-bone interface using allograft, cement, expandable features, and hydroxyapatite coating. However, these attempts have not translated into improved clinical outcomes [10, 11]. There is a continued need for fixation options that provide the immediate stability of pedicle screw constructs without the associated failure modes.

There has been growing interest in using computational methods such as finite element analysis (FEA) to enhance the understanding of the biomechanical characteristics of spinal implants [12–14]. Unlike clinical studies that rely on patient outcomes and postoperative observations, FEA allows researchers to simulate and analyze the stress, strain, and displacement patterns within the spine and implant components. By precisely controlling the simulation parameters, FEA enables investigations into the potential causes of implant failure, loosening, and complications that may be difficult to explore in a clinical study. Furthermore, FEA facilitates comparative analyses to directly compare the biomechanical effects between different implant designs, implant locations, or surgical approaches [15].

The current FEA study evaluated an FDA-cleared polyetheretherketone (PEEK) strap that provides circumferential cortical fixation by utilizing the strongest elements of the posterior lumbar spine (lamina, superior articular process, and inferior articular process), which are anchored to the pedicle. Using a traditional open or minimally invasive approach, the cortico-pedicular posterior fixation (CPPF) device is implanted through two intersecting bone tunnels in the posterior column, providing immediate stability during lumbar fusion procedures. A hypothetical advantage of this device is the potential for anterior load transfer, which may enhance fusion rates by promoting bone growth and facilitating the incorporation of interbody devices [16]. This study aimed to compare the biomechanical characteristics of stability and load-sharing between the CPPF device and a conventional pedicle screw system (PSS) in different instrumentation scenarios using FEA.

## Materials and methods

### Study design

**Fig. 1:**
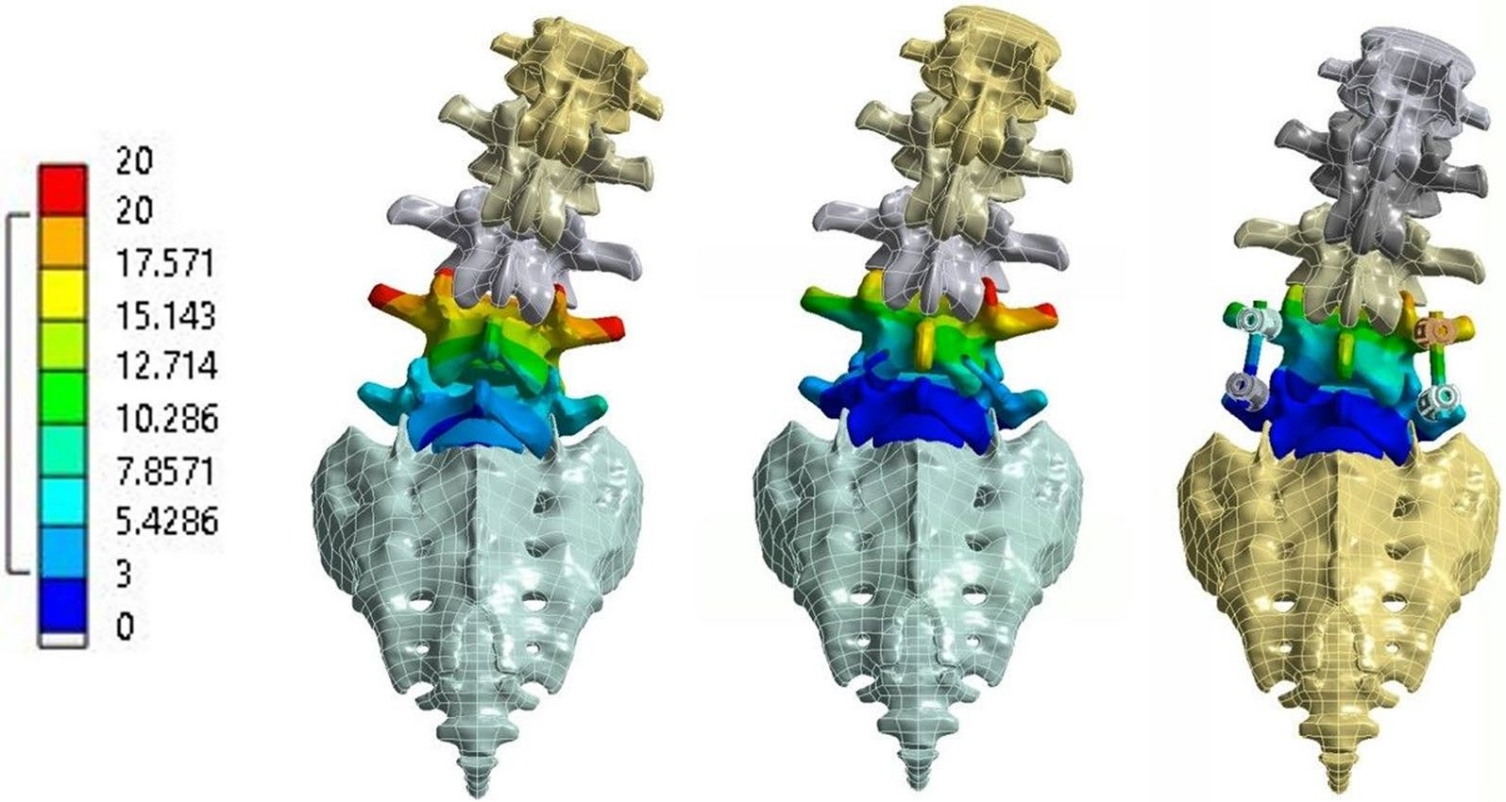
L4–S1 spine, color scale (in mm) shows the deformation on lateral bending for the intact spine (left), cortico-pedicular posterior fixation (CPPF) device (middle), and pedicle screw system (PSS) (right)

Multiple FEAs were performed to compare the biomechanical characteristics of the CPPF device (Karma Posterior Fixation Device, Spinal Elements, Carlsbad, CA, USA) to a PSS. Three different instrumentation strategies of the lumbar spine were considered: (a) single-level LLIF with CPPF or PSS, (b) two-level LLIF with CPPF or PSS, and (c) single-level ALIF with CPPF or PSS. Depending on the instrumented levels, two different interbody devices were used: lateral lumbar interbody fusion (LLIF) or anterior lumbar interbody fusion (ALIF) (Table 1; Fig. 1). An uninstrumented healthy lumbar spine was also evaluated as a reference for the different output metrics.

**Table 1:** Finite element analysis instrumentation simulation conditions

Instrumented levels	Interbody device	Instrumentation evaluated
Uninstrumented	No device	No device
L4–L5	LLIF	CPPF and PSS
L5-Sacrum	ALIF	CPPF and PSS
L4-Sacrum	ALIF	CPPF and PSS

*ALIF*—anterior lumbar interbody fusion, *CPPF*—cortico-pedicular fixation, *LLIF*—lateral lumbar interbody fusion, *PSS*—pedicle screw system

### Healthy spine finite element model

**Fig. 2:**
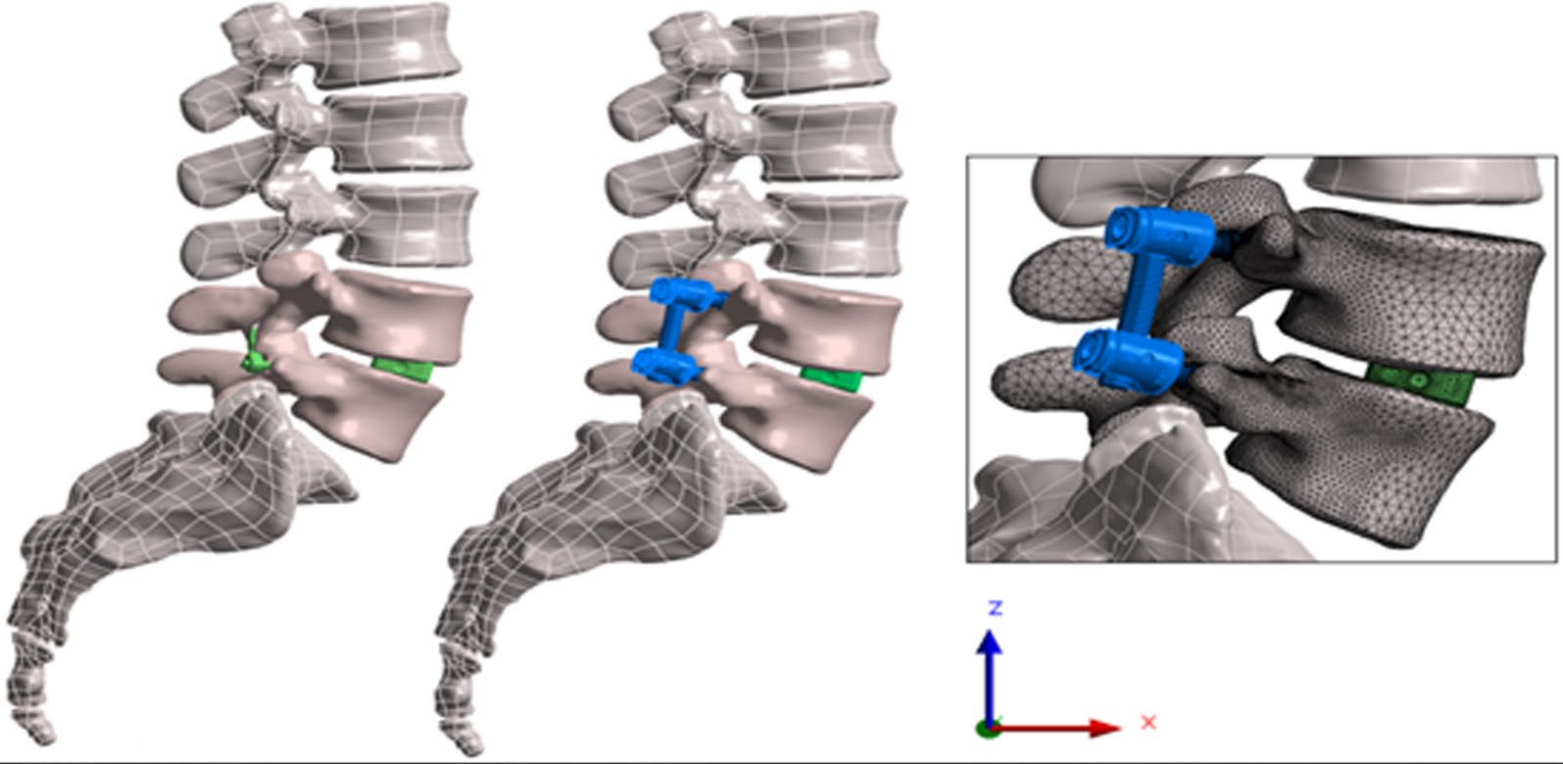
L4–L5 cortico-pedicular posterior fixation (CPPF) device and pedicle screw system (PSS)-instrumented spine model, with an example of bone and instrumentation meshing detail

An osteoligamentous finite element model of the lumbar spine (L1 to sacrum) was built based on three-dimensional geometry reconstructed from computed tomography imaging (Zygote, American Fork, UT, USA). Based on the healthy spine model, a second model was adapted to represent a degenerative disk condition with reduced lordosis (− 7°) and intervertebral space (− 25% at the endplate center). A spinal axis system was defined with a posteroanterior *x*-axis, a mediolateral *y*-axis, and a *z*-axis along the vertical gravity line (Fig. 2) using Ansys 2022R1 FEA software (Ansys, Canonsburg, PA, USA).

**Fig. 3:**
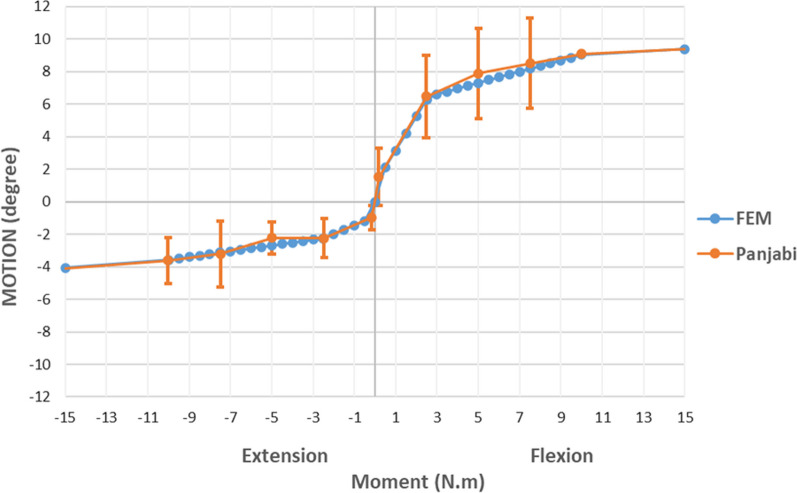
Comparison of the moment-rotation curves between Panjabi data [27] and the finite element model (FEM) for the extension-flexion stiffness of the spinal functional unit L4–L5

The modeling characteristics of similar models have been described in previous publications [17–22] and are summarized hereafter. The instrumented vertebrae were modeled as flexible bodies, including a cancellous bone core and a cortical bone layer, while the noninstrumented vertebrae were modeled as rigid bodies. The cortical layer thicknesses were modeled as 1.6 mm for vertebrae posterior elements, 0.5 mm for the vertebral body, and 1.25 mm for the sacrum. Young’s modulus was assigned as 241 MPa for cancellous bone and 1091 MPa for cortical bone. The stiffness contribution of the soft tissues of each functional spinal unit (FSU), which consisted of two adjacent vertebrae, the intervertebral disk, and all adjoining ligaments and articular facets, was modeled using nonlinear three-dimensional springs, represented by a 6 × 6 stiffness matrix. Each translational and rotational coefficient of the stiffness matrix captures the nonlinear load‒displacement (moment-rotation) movement as reported within the literature, where each segmental unit’s biomechanical behavior was derived from cadaveric studies [23–27]. An example of this calibration process outcome is illustrated in Fig. 3 for FSU L4–L5 in flexion–extension.

### Spinal instrumentation finite element model

The FSU stiffness was modified to account for ligament resection associated with the surgical techniques. The entire nucleus pulposus was removed for all instrumented levels along with 40% of the annulus fibrosis (AF) and the anterior ligament for ALIF cage surgery and 20% of the AF for LLIF. The sizes and positionings of the different posterior fixation and interbody devices were assigned based on clinical feedback from the coauthors (RE, PN, AV). The Young modulus was set to 4.1 GPa for PEEK devices (CPPF and interbody devices, data provided by device manufacturer) and to 113.8 GPa for Ti6Al4V devices (screws and rods). Frictional contact with a coefficient of friction (CoF) of 0.2 was used for the interfaces between screws and bone and between the CPPF device and bone. The bone channels for CPPF device fixation were modeled with contoured, rounded edges to reflect clinical surface contact between the device and bone. A CoF of 0.46 was used to account for the decorticated facet joint interface in CPPF device models [28]. The interbody device and vertebral endplate contact interface was defined as rough, i.e., infinite CoF, to simulate the effect of teeth on the interbody devices.

The ALIF and LLIF cages were selected in consultation with practicing spine surgeons to match the anatomy of the specific spinal segments modeled. The ALIF cage had integrated titanium screws (5.5 × 25 mm), 41 mm lateral length, 10 mm (L4–L5) or 11 mm (L5–S1) height, 27 mm anterior–posterior length, 1107 mm^2^ footprint area, 12° lordosis, and porous titanium-coated PEEK. The LLIF cage had no integrated screws, 40 mm lateral length, 7 mm height, 22 mm anterior–posterior length, 880 mm^2^ footprint area, 6° lordosis, and porous titanium-coated PEEK. Both cages were modeled in a neutral position without techniques to further increase lordosis.

For the CPPF model, the facet joints were decorticated per the device’s instructions for use to enable fixation and fusion. The PSS model maintained intact facet joints to allow posterior force transfer, as partial decortication would require arbitrary posterior compression that could confound results. This allowed standardized comparison of the posterior load shift due to the metal rod stiffness without additional posterior forces.

### Simulation process

A follower load of 415 N, representing the combined effect of gravity and the associated muscle stabilization, was calculated for a 90 kg patient and applied on each vertebral cranial endplate. A node positioned at the center of the L1 cranial endplate was connected using a multipoint constraint to the endplate nodes. The different functional movements were simulated by applying a moment to this node and restricting displacements and rotations as described in Table 2. For all scenarios, the lower half section of the sacrum was fixed in space.

**Table 2:** Boundary conditions applied on the L1 endplate

Functional movement	Moment	L1 fixed displacements and rotations
Flexion	*My* = 7.5 Nm	Y, RX, RZ
Extension	*My* = −7.5 Nm	Y, RX, RZ
Lateral bending (right)	*Mx* = 7.5 Nm	X^†^, RY^†^, RZ
Torsion (left)	*Mz* = 7.5 Nm	X^†^, Y, RX, RY^†^
Weight only	No moment	X^†^, Y, RX, RZ

^†^Displacements are fixed after device installation

The following simulation steps were performed for all instrumented models. For the PSS, screws were inserted into the vertebrae. For interbody device insertion, the device was positioned, and contact was activated to reach a force equilibrium state. For posterior fixation of the PSS, the rod was rigidly attached to the screws, which were inserted into the vertebrae before simulation. For CPPF, the geometry was adapted to its installed final shape, the device was positioned into predrilled holes in vertebrae and meshed with solid elements, contact with vertebrae was activated, and the device was placed under tension by application of a 222 N preload representative of a normal installation. Finally, the application of follower load and functional motions were simulated. The FSU range of motion (ROM) was measured as the total rotation between the FSU upper and lower endplates at the maximum applied moment. This allows comparison to the stabilization performance of the CPPF device with the PSS. Anterior load transfer was calculated as the total load through the disk and interbody device after functional motion and follower load application to compare the load-sharing characteristics between the two systems.

## Results

**Fig. 4:**
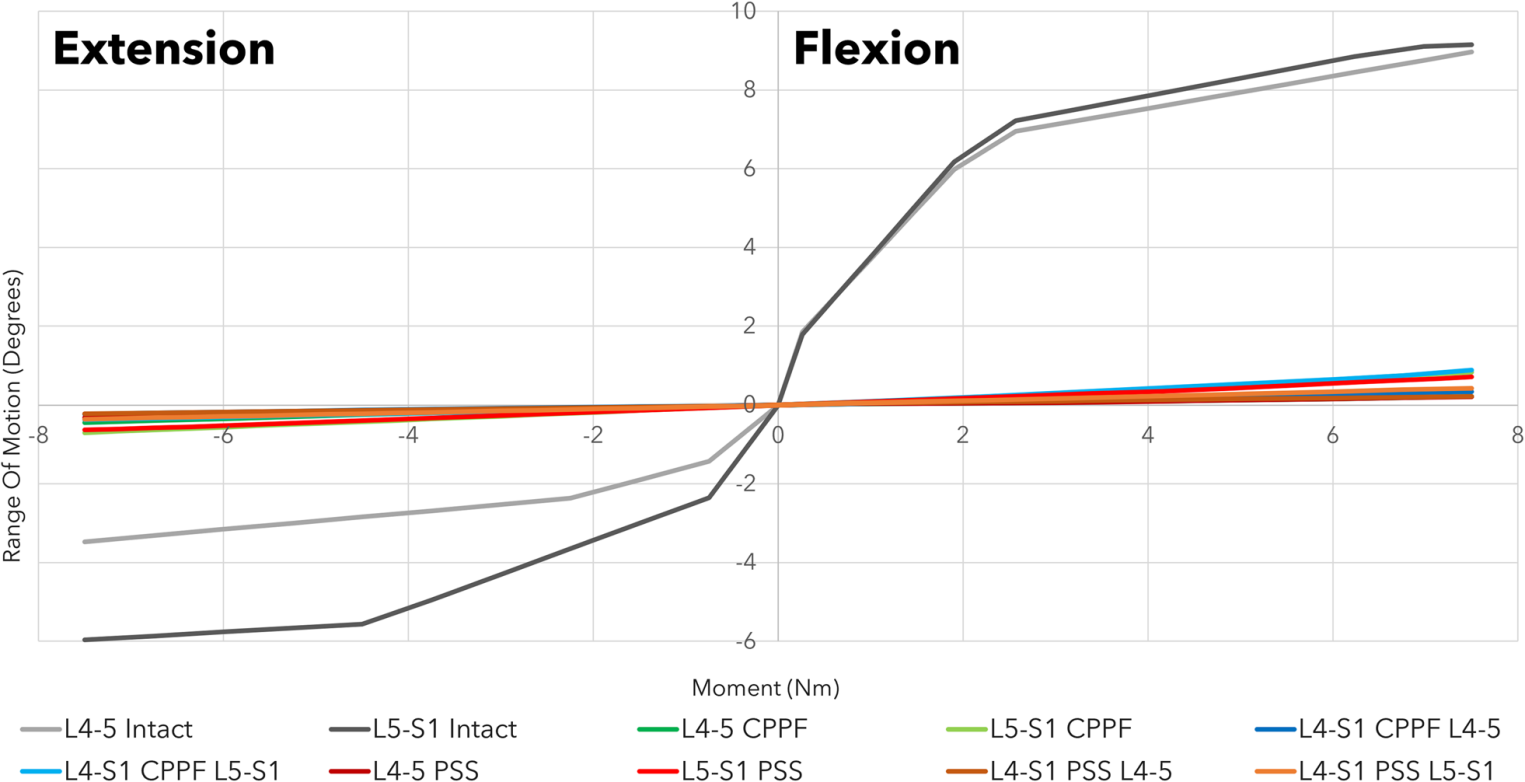
Moment-rotation curve in flexion–extension with the cortico-pedicular posterior fixation (CPPF) device, pedicle screw system (PSS), and intact spine at one or two operated segments. At a pure moment of 7.5 Nm, flexion–extension between the CPPF device and PSS was within 0.5 degrees under all loading conditions

**Fig. 5:**
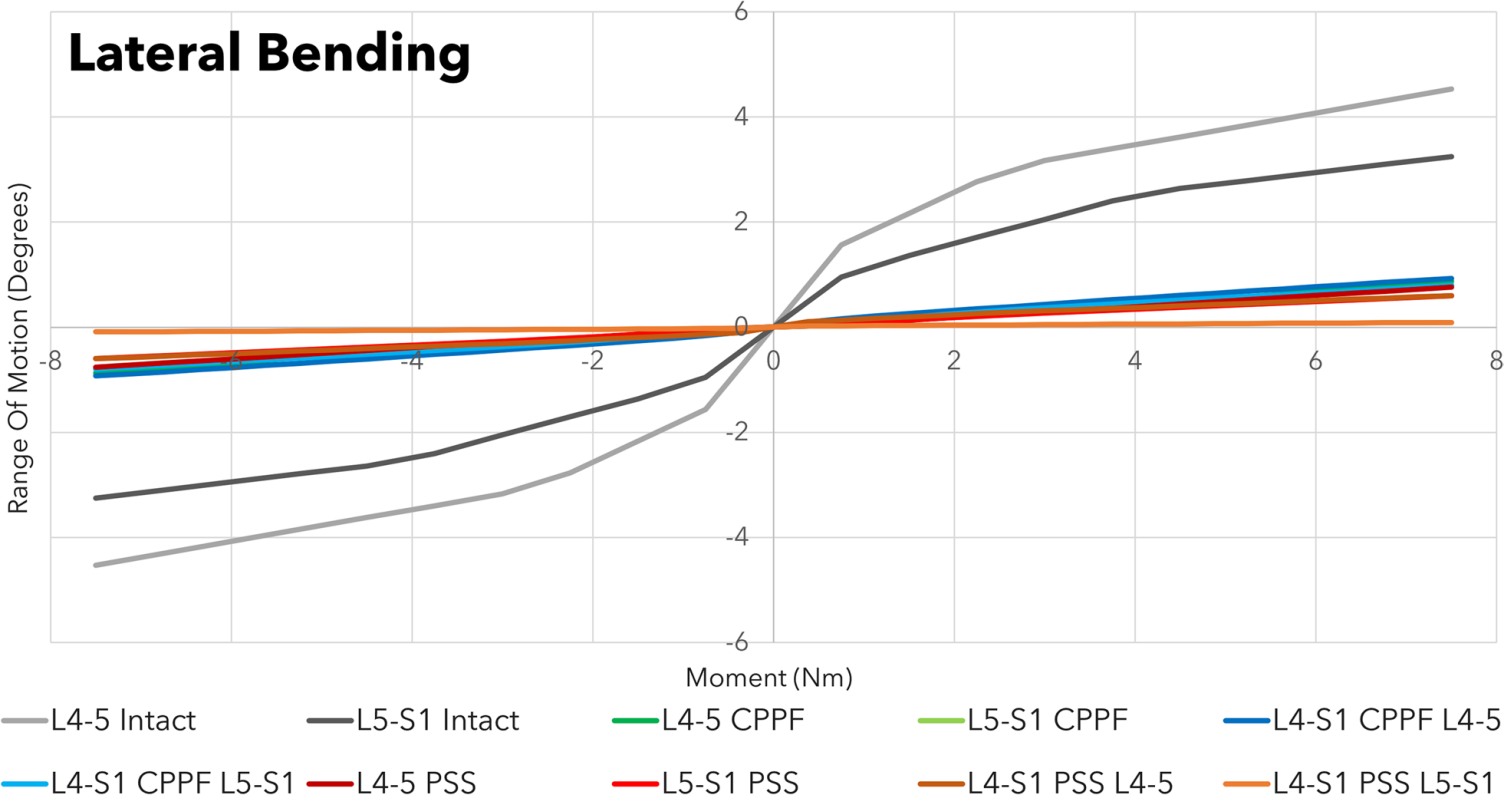
Moment-rotation curve in lateral bending with the cortico-pedicular posterior fixation (CPPF) device, pedicle screw system (PSS), and intact spine at one or two operated segments. At a pure moment of 7.5 Nm, lateral bending between the CPPF device and PSS was within 0.6 degrees under all loading conditions

**Fig. 6:**
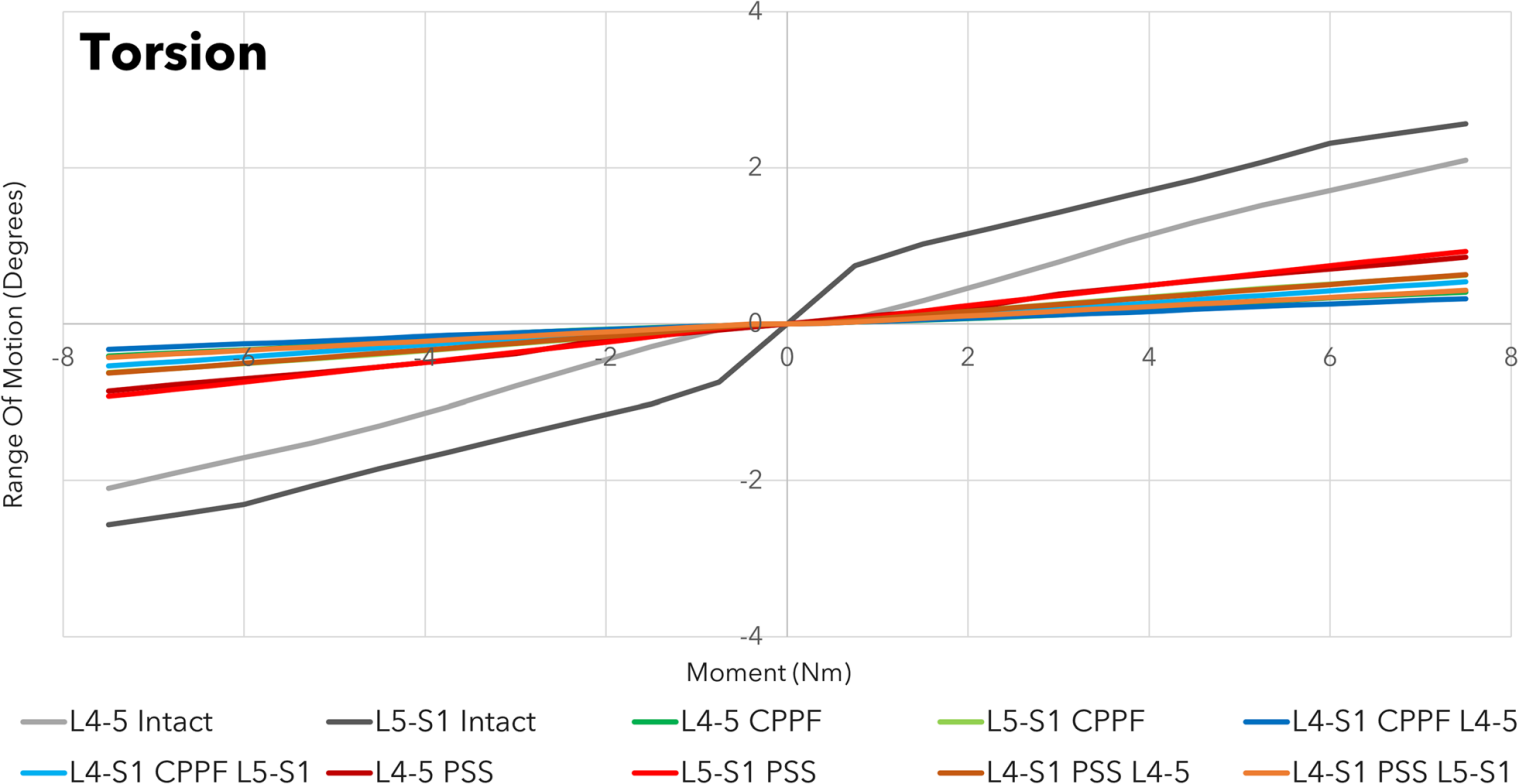
Moment-rotation curve in torsion with a cortico-pedicular posterior fixation (CPPF) device, pedicle screw system (PSS), and intact spine at one or two operated segments. At a pure moment of 7.5 Nm, torsion between the CPPF device and PSS was within 0.5 degrees under all loading conditions

**Fig. 7:**
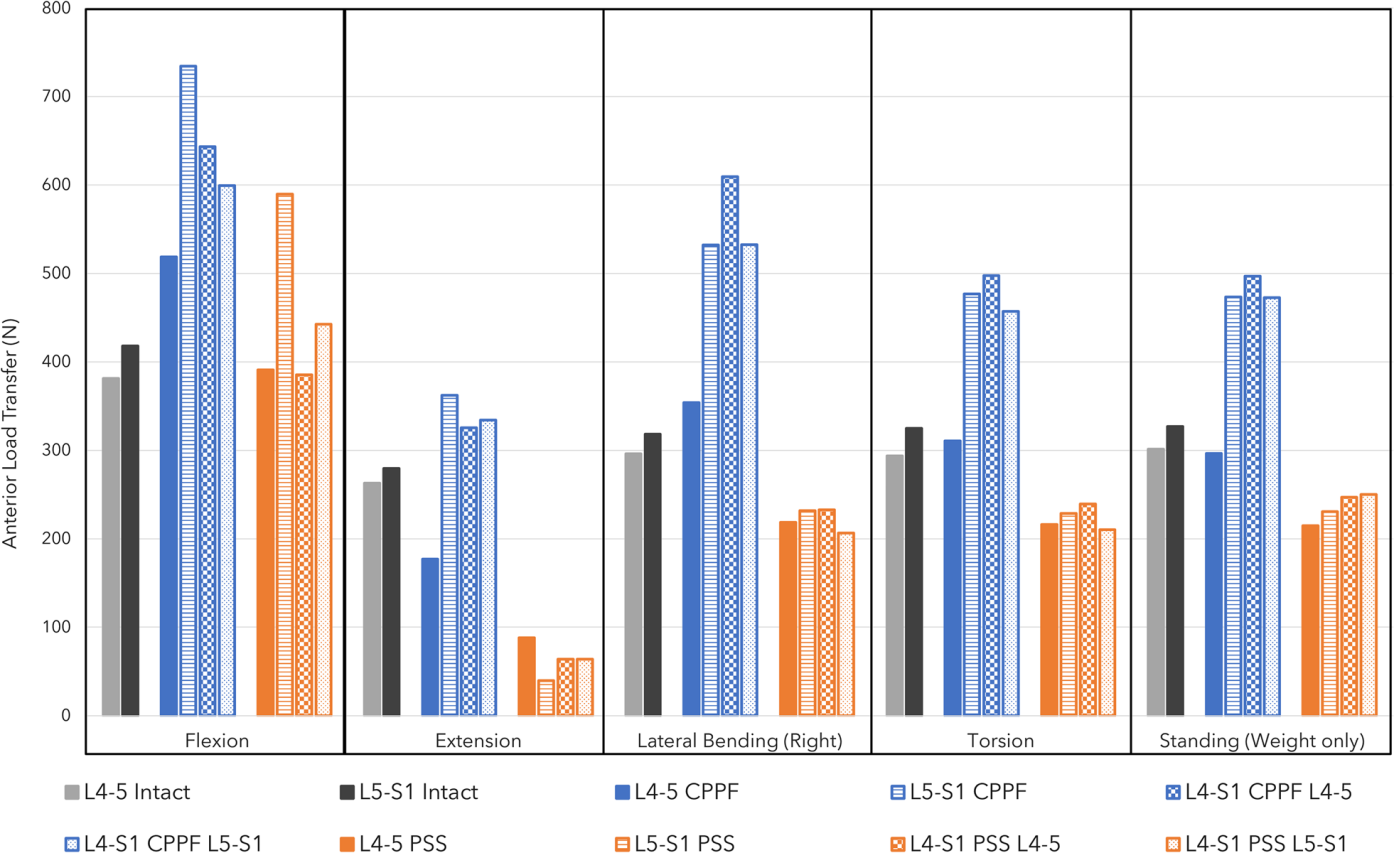
Total anterior load transfer with a cortico-pedicular posterior fixation (CPPF) device, pedicle screw system (PSS), and intact spine. Anterior loading between PSS and CPPF is reported across four instrumented spinal levels from three separate finite element analysis models including single-level lateral lumbar interbody fusion (LLIF) with PSS or CPPF (1 level), two-level LLIF with PSS or CPPF (2 levels), and single-level anterior lumbar interbody fusion (ALIF) with PSS or CPPF (1 level)

**Fig. 8:**
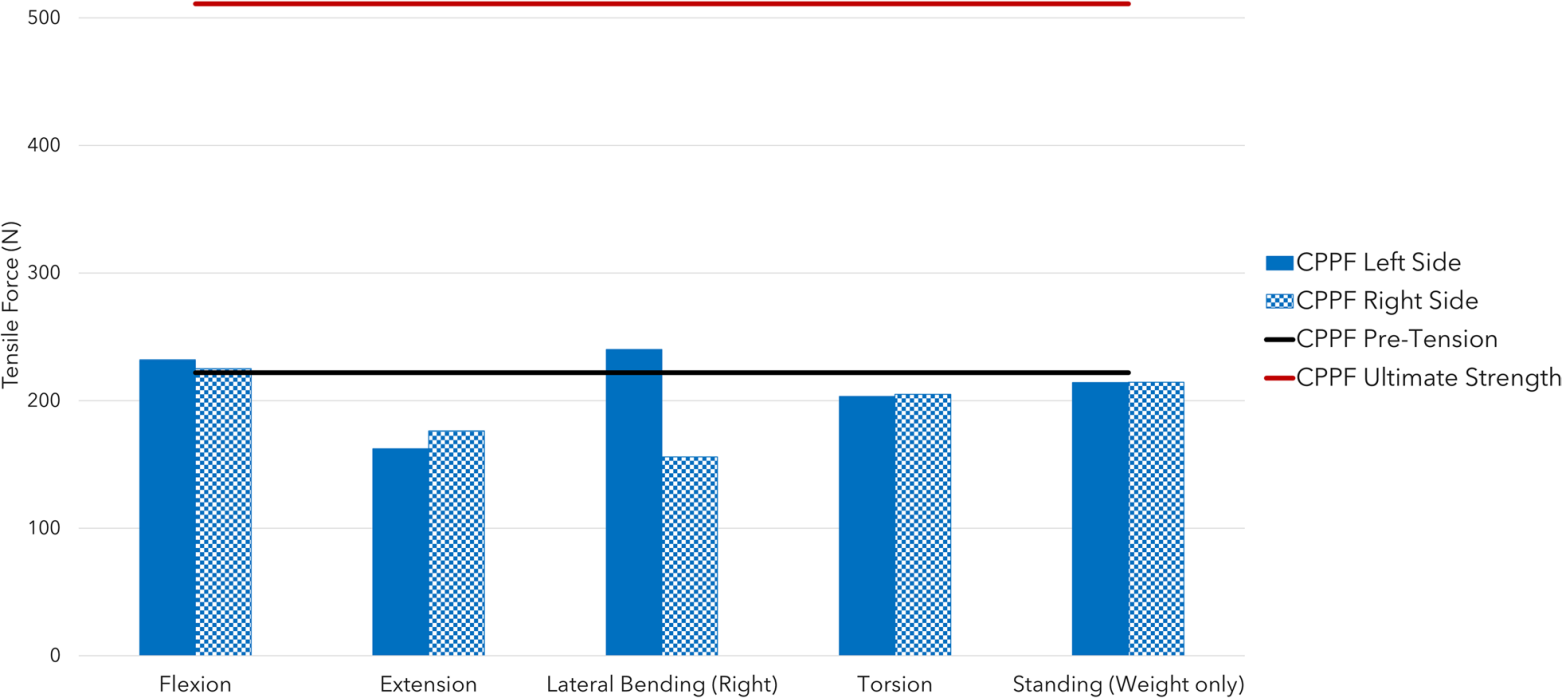
Cortico-pedicular posterior fixation (CPPF) device tensile force at maximal loading under flexion, extension, standing, torsion, and right lateral bending

Across all combinations of interbody devices and lumbar levels evaluated, CPPF consistently demonstrated significant reductions in flexion (ranging from 90 to 98%), extension (ranging from 88 to 94%), lateral bending (ranging from 75 to 80%), and torsion (ranging from 77 to 86%) compared to the intact spine. The stability provided by CPPF was comparable to that of PSS in all simulations (Table 3). Specifically, with a 7.5 Nm moment, the differences in ROM between CPPF and PSS were negligible, ranging from 0.5 degrees for flexion–extension (Fig. 4), 0.6 degrees for lateral bending (Fig. 5), and 0.5 degrees for torsion (Fig. 6). All ROM values for both CPPF and PSS were less than 1 degree in all simulations, which is consistent with previous studies using PSS in the lumbar spine [29]. In addition, the CPPF demonstrated greater anterior load transfer than the PSS under various simulated conditions. Comparing CPPF to PSS across all tested conditions, the difference in anterior load transfer ranged from 128 to 258 N during flexion, 89–323 N during extension, 135–377 N during lateral bending, 95–258 N during torsion, and 82–250 N during standing (Fig. 7). Under all simulated conditions, the tensile force experienced by the CPPF device remained below 50% of its 487 N yield load, suggesting a low probability of device failure under normal loading conditions (Fig. 8).

**Table 3:** Range of motion with three models at four operated segments at a pure moment of 7.5 Nm

Motion	Lumbar level	Implant location	ROM (°)			Difference in ROM (°)		
			Intact spine	CPPF	PSS	PSS versus intact spine	CPPF versus intact spine	CPPF versus PSS
Flexion	L4–L5	L4–L5	9.0	0.2	0.2	− 8.8	− 8.8	0.0
	L5–S1	L5–S1	9.2	0.8	0.7	− 8.5	− 8.4	0.1
	L4–S1	L4–L5	9.0	0.3	0.2	− 8.8	− 8.7	0.1
	L4–S1	L5–S1	9.2	0.9	0.4	− 8.8	− 8.3	0.5
Extension	L4–L5	L4–L5	− 3.5	− 0.4	− 0.3	3.2	3.1	− 0.1
	L5–S1	L5–S1	− 6.0	− 0.7	− 0.6	5.4	5.3	− 0.1
	L4–S1	L4–L5	− 3.5	− 0.2	− 0.2	3.3	3.3	0.0
	L4–S1	L5–S1	− 6.0	− 0.4	− 0.4	5.6	5.6	0.0
Lateral bending	L4–L5	L4–L5	4.5	0.9	0.8	− 3.7	− 3.6	0.1
	L5–S1	L5–S1	3.2	0.8	0.6	− 2.6	− 2.4	0.2
	L4–S1	L4–L5	4.5	0.9	0.6	− 3.9	− 3.6	0.3
	L4–S1	L5–S1	3.2	0.7	0.1	− 3.1	− 2.5	0.6
Torsion	L4–L5	L4–L5	2.1	0.4	0.9	− 1.2	− 1.7	− 0.5
	L5–S1	L5–S1	2.6	0.6	0.9	− 1.7	− 2.0	− 0.3
	L4–S1	L4–L5	2.1	0.3	0.6	− 1.5	− 1.8	− 0.3
	L4–S1	L5–S1	2.6	0.5	0.4	− 2.2	− 2.1	0.1

*CPPF*—cortico-pedicular fixation*, PSS*—pedicle screw system, *ROM*—range of motion

## Discussion

This study utilized FEA to evaluate the biomechanical performance of the CPPF device in comparison to PSS in multiple lumbar fusion scenarios, including single-level LLIF, two-level LLIF, and single-level ALIF. Our findings revealed that supplemental fixation with the CPPF device decreased ROM by 75% to 98% compared to the intact spine and provided stability comparable to PSS with higher anterior load transfer. These results align with previous research highlighting the importance of spinal stabilization in reducing postoperative pain [30]. The higher anterior load transfer observed in our study is also noteworthy, as it may enhance fusion rates by promoting bone growth and facilitating the incorporation of interbody devices [16, 31]. Overall, the results of this FEA highlight the potential clinical utility of the CPPF device as a viable alternative to PSS in lumbar fusion surgeries.

The CPPF device used in this FEA biomechanical study is commercially available for cortico-pedicular posterior fixation during lumbar fusion procedures. The fixation provided by the cortico-pedicular strap allowed for load transfer through the facet joint instead of the alternate route of bilateral rods in PSS. This avoids the phenomenon of stress shielding observed with metal alloys used in most PSSs with mechanical lever arms extending beyond the instantaneous axis of rotation. Consequently, the tensile forces within the strap remained near the pretension level of 222 N in all loading modes. While this FEA study demonstrated biomechanical advantages of the CPPF device, future clinical studies should examine its influence on fusion rates, clinical outcomes, and adjacent segment degeneration compared to traditional PSS.

Pedicle screw construct stability relies on anchorage with bone, and failure rates of screw loosening, pullout, and migration are not insignificant [7]. Pedicle screw anchorage failure rates are considerably higher in osteoporotic patients [32, 33]. Therefore, many patients are unsuitable candidates for traditional metal fixation devices due to low bone quality, increasing the risk of screw pullout. The CPPF device may potentially overcome this limitation by providing circumferential fixation via higher-density cortical bone of the pedicle neck, lamina, and facet joints.

Finite element analysis has been increasingly utilized to investigate the biomechanical properties of posterior fixation techniques [12–14], providing several unique advantages in the current study. With FEA, the structural geometry, material properties, and load boundary conditions of the spine were simulated by using mathematical models to measure the biomechanical impact of changing relevant parameters on the overall structure. The FEA model was particularly advantageous since it allowed for cause‒effect relationships to be isolated and fully explored. However, the FEA presented in this study also had a few limitations that warrant further discussion. First, the FEA models introduced several simplifications to minimize model complexity and ensure convergence in a reasonable amount of time. Nevertheless, validation of the FEA model with ROM in an intact spine showed that segmental flexibility compared favorably with prior studies, suggesting that the model simplifications likely had minimal influence on data interpretation. Second, lumbar spine biomechanical properties vary between individuals; therefore, the models in the present study could not account for individual biological variations or the effects of surrounding muscles and soft tissues. Finally, translation of biomechanical data from FEA simulations to clinical outcomes in patients with degenerative lumbar disease must be confirmed in prospective studies.

## Conclusions

Under the modeled conditions, cortico-pedicular fixation for supplementing anterior or lateral interbody devices between L4 and S1 resulted in comparable stability based on ROM measures and less anterior column stress shielding based on total anterior load transfer measures compared to PSS. These results suggest that the CPPF device may represent a promising alternative to PSS for supplemental fixation during lumbar fusion. Clinical studies are needed to confirm these FEA findings.

## Data Availability

The datasets used and/or analyzed during the current study are available from the corresponding author on reasonable request.
